# Stakeholder Perspectives on Implementing a Firearm Safety Intervention in Pediatric Primary Care as a Universal Suicide Prevention Strategy: A Qualitative Study

**DOI:** 10.1001/jamanetworkopen.2018.5309

**Published:** 2018-11-30

**Authors:** Courtney Benjamin Wolk, Amelia E. Van Pelt, Shari Jager-Hyman, Brian K. Ahmedani, John E. Zeber, Joel A. Fein, Gregory K. Brown, Courtney A. Gregor, Adina Lieberman, Rinad S. Beidas

**Affiliations:** 1 Department of Psychiatry, Perelman School of Medicine, University of Pennsylvania, Philadelphia; 2 Leonard Davis Institute of Health Economics, University of Pennsylvania, Philadelphia; 3 Department of Biostatistics, Epidemiology, and Informatics, Perelman School of Medicine, University of Pennsylvania, Philadelphia; 4 Henry Ford Health System, Center for Health Policy & Health Services Research and Behavioral Health Services, Detroit, Michigan; 5 Center for Applied Health Research, Baylor Scott & White Health, jointly with Central Texas Veterans Health Care System, Temple; 6 School of Public Health, University of Massachusetts at Amherst, Amherst; 7 Division of Emergency Medicine, The Children’s Hospital of Philadelphia, Philadelphia, Pennsylvania; 8 Department of Pediatrics, Perelman School of Medicine, University of Pennsylvania, Philadelphia; 9 Department of Medical Ethics and Health Policy, Perelman School of Medicine, University of Pennsylvania, Philadelphia

## Abstract

**Importance:**

The rate of youth suicide has increased steadily over the past several decades due, in part, to an increase in suicide by firearm. Implementation of evidence-based approaches to increase safe firearm storage practices are important for reducing youth suicide.

**Objective:**

To assess the needs of stakeholders who would be affected by implementing an evidence-based approach to firearm safety promotion—Firearm Safety Check, which includes screening for the presence of firearms in the home, brief motivational interviewing–informed counseling regarding safe firearm storage, and provision of free firearm locks—in pediatric primary care settings.

**Design, Setting, and Participants:**

In this qualitative study, 58 stakeholders were interviewed over a 7-month period across 9 stakeholder groups from 2 large and diverse health systems. Participants included parents of youth; physicians; nurses and nurse practitioners; leaders of pediatric primary care practices, behavioral health, and quality improvement; system leaders; third-party payers; and members of national credentialing bodies. Data analysis were conducted from September 2017 to April 2018.

**Main Outcomes and Measures:**

Interview guides were informed by the Consolidated Framework for Implementation Research. An integrated analysis approach was used in which a priori attributes of interest were identified (Consolidated Framework for Implementation Research constructs, eg, intervention characteristics), and an inductive approach was used with regard to new themes that emerged.

**Results:**

A total of 58 stakeholders were interviewed (mean [SD] age, 48.38 [10.65] years; 27 men and 31 women). Stakeholders indicated that firearm safety promotion is a health system priority and reported favorable perceptions of the Firearm Safety Check approach for suicide prevention. The importance of leveraging existing infrastructures, such as electronic health record systems and brevity, were frequently noted. Distribution of firearm locks was endorsed as the most complicated component to implement.

**Conclusions and Relevance:**

By including multiple service system stakeholder perspectives, these results support the development of implementation strategies to change clinician, organization, and system behaviors around firearm safety promotion in pediatric primary care as a universal suicide prevention strategy.

## Key Points

**Question:**

What are the needs of stakeholders who would be affected by implementing an evidence-based approach to firearm safety promotion in pediatric primary care as a universal suicide prevention strategy?

**Findings:**

In this qualitative study of 58 stakeholders, firearm safety promotion for suicide prevention was described as acceptable, mostly feasible to implement, and a health system priority. Participants described the importance of leveraging existing infrastructures and brevity.

**Meaning:**

The results of this contextual inquiry provide valuable insights toward future efforts to implement firearm safety promotion in pediatric primary care to prevent suicide.

## Teaser

This qualitative study assesses the needs of stakeholders who would be affected by implementing an evidence-based approach to firearm safety promotion in pediatric primary care settings as a youth suicide prevention strategy.

## Introduction

Youth suicide is the second leading cause of death among people aged 10 to 24 years.^1^ Firearms are the most frequently used and most lethal means. Risk of suicide is 2 to 5 times greater in homes containing firearms; 1 in 3 US homes contain a firearm.^2,3^ To date, suicide prevention strategies have been implemented in behavioral health settings even though only one-third of youths who attempt suicide receive mental health treatment in the 12 months prior.^4,5^ In contrast, more than 75% of youths who die by suicide visit pediatric primary care in the year prior.^6^ Thus, suicide prevention strategies limited to behavioral health settings are insufficient, and research on how to optimize primary care for suicide prevention is warranted.

Firearm safety promotion is a promising suicide prevention strategy that can be implemented in pediatric primary care. Safety Check is one evidence-based approach that has been associated with safer parental firearm storage in youths aged 2 to 11 years.^7^ Originally, the firearm component of Safety Check was bundled with other strategies (eg, reduced screen time) and included screening for firearms in the home, brief motivational interviewing–informed counseling regarding safe storage, and provision of free firearm locks. In a cluster randomized trial of 137 pediatric primary care practices, parents who received Safety Check were 21% more likely to engage in safe gun storage after 6 months compared with parents in the control arm.^7^

There is clear consensus that promoting firearm safety in pediatric primary care is essential in reducing youth suicide.^8^ Physician groups, including the American Academy of Pediatrics^9^ and the National Academy of Medicine,^10^ recommend that physicians discuss firearm safety with parents. However, physicians remain reluctant to discuss firearm safety in primary care, and the 3 firearm-related components of Safety Check (ie, screening, brief counseling, and distribution of firearm locks, which we refer to as the Firearm Safety Check intervention to distinguish it from the full Safety Check intervention described above) remain underused (R.S.B., unpublished data, 2016).^11^

To gain a better understanding of why firearm safety promotion as a universal suicide prevention strategy remains underused in pediatric primary care, it is important to understand the implementation context.^12^ The Consolidated Framework for Implementation Research (CFIR)^13^ provides a framework to better understand the perspectives of stakeholders who would be affected by such an intervention. The 5 major CFIR domains are as follows: (1) intervention characteristics (ie, the features of the intervention), (2) outer setting (ie, the economic, political, and social context within which an organization exists), (3) inner setting (ie, the organizational setting), (4) characteristics of individuals involved in implementation, and (5) the implementation process. To bring firearm safety promotion to scale, it is necessary to first understand barriers and facilitators across these 5 domains. It is of particular importance to conduct contextual inquiry given that discussing firearm-related matters in health care has been controversial and a topic of legislation (eg, the Privacy of Firearm Owners bill).^14^ Firearm safety interventions focused on suicide may involve determinants distinct from other safety interventions, such as the use of car seats.^15^

To our knowledge, no studies to date have systematically identified factors that may hinder or enable implementation of firearm safety promotion practices as a suicide prevention strategy in pediatric primary care. Information yielded from multiple stakeholder groups can enable the development of precise implementation strategies and maximize the likelihood of bringing the intervention to scale.^16^ The primary aim of this study was to use a qualitative approach to examine barriers and facilitators and supports needed to effectively implement the Firearm Safety Check intervention. We solicited the perspectives of stakeholders across 2 large, diverse, and geographically distinct health systems, including a subset of firearm-owning clinicians, leaders of pediatric primary care practices, and parents.

## Methods

### Participants

Stakeholders from 9 stakeholder groups participated: parents of youth ages 12 to 24 years, physicians, nurses and nurse practitioners, leaders of pediatric primary care practices, leaders of behavioral health (given the need for coordination between behavioral health and primary care if Firearm Safety Check would be implemented at scale and given integrated behavioral health practices in both health systems), leaders of quality improvement, system leaders, third-party payers, and members of national credentialing bodies. Participants were recruited via telephone and email from a large Midwestern health system and a large Southern health system and nationally (ie, third-party payers and national credentialing bodies) (Table 1). Purposive sampling was conducted to recruit 4 to 7 individuals from each stakeholder group, and stratified sampling was conducted for physicians and leaders of pediatric primary care practices to ensure endorsement of the use of at least 1 of the Firearm Safety Check components (based on data obtained from a previous survey) so that participants would have experience with intervention strategies (R.S.B., unpublished data, 2016). Firearm ownership information was collected from leaders of pediatric primary care practices, physicians, and parents. All procedures were approved by the institutional review board at the University of Pennsylvania. Two additional health system institutional review boards were involved; 1 engaged in a reliance agreement with the institutional review board at the University of Pennsylvania, the other granted exemption. Oral informed consent was obtained from all participants, and no one withdrew from the study following informed consent. We followed the Consolidated Criteria for Reporting Qualitative Research (COREQ) reporting guideline.^17^

**Table 1.** Participant Inclusion Criteria

Stakeholder Group	Eligibility Criteria
Parents of youth	English-speaking guardians of youths aged 12-24 y who visited a primary care site within the previous 3 mo, as determined by their electronic health records
Physicians	A physician in pediatric primary care who participated in a previous study survey and endorsed usage of ≥1 firearm safety check component
Nurses and nurse practitioners	An appropriate individual who was identified as a pediatric primary care nonphysician clinician (ie, nurse or nurse practitioner) by an investigator, a leader of practices, or a pediatric primary care physician
Leaders of pediatric primary care practices	An individual who was identified as a physician leader of a pediatric primary care clinic (eg, medical director) who participated in a previous study survey
Leaders of behavioral health	A behavioral health department leader who was identified by an investigator at either site
Leaders of quality improvement	An appropriate individual involved with quality improvement who was identified by an investigator at either site
System leaders	An appropriate health system leader who was identified by an investigator at either site
Third-party payers	A representative of a private or public insurer
Members of national credentialing bodies	A representative of a national credentialing body or relevant national clinician group

### Procedure

Informed by the CFIR, semistructured interview guides were developed and piloted. The beginning of the interview provided information on the effectiveness of the intervention, citing the original trial. Interview guides included questions about interviewees’ perception of the role of pediatric primary care in suicide prevention, the culture around firearms in the communities served by the health systems, current use of the 3 components of Firearm Safety Check, acceptability and feasibility of these components, barriers and facilitators to implementation, and perspectives about who should implement various components. The focus was on the Firearm Safety Check as a universal suicide prevention strategy. For physicians, leaders of practices, and system leaders, aggregated data on their health system’s current use of Firearm Safety Check components and acceptability of the intervention, derived from a previous survey of physicians and leaders of pediatric practices, were presented during the interview and feedback was elicited (R.S.B., unpublished data, 2016).

The 1-time interviews were conducted by 6 University of Pennsylvania study staff and investigators. Interviewers had no previous relationships with interviewees. During the consent process, the intentions of the research team were shared. Interviews were conducted privately over the telephone, audio recorded, and lasted on average 1 hour. No field notes were recorded. The interviewers met regularly to discuss thematic saturation.

Each participant received $25 for their participation. Participants endorsed ethnicity and race using the categories recommended for federal data collection purposes and requested by the National Institutes of Health. All interviews were transcribed and uploaded into the NVivo Qualitative Data Analysis Software, version 10 (QSR International) program for storage and management. Transcripts were not shared with participants, nor did participants provide feedback on findings.

### Data Analysis

An inductive process of iterative coding was used to ascertain recurrent relationships, themes, and categories. We used an integrated analysis approach,^18^ identifying a priori attributes (CFIR constructs), and also used modified grounded theory.^19,20^ Three members of the research team (C.B.W., A.E.V.P., and C.A.G.) separately coded 3 transcripts and compared applications of the coding system with assess robustness and reliability. Disagreements were resolved through team discussion and the codebook was refined (available on request). The revised codebook was then applied to all interviews. All interviews were coded by one of us (A.E.V.P.), and another (C.A.G.) double coded 20% (κ = 0.87). On completion of the analyses, a post hoc analysis was conducted to determine differences in themes between firearm-owning (n = 7) and non–firearm-owning (n = 14) stakeholders and between parents (n = 7) and other stakeholder groups (n = 51). However, owing to the small sample size within the subgroups, saturation of themes may not have been achieved.

## Results

A total of 58 stakeholders were interviewed (mean [SD] age, 48.38 [10.65] years; 27 men and 31 women) using the 5 major CFIR domains: (1) intervention characteristics (ie, the features of the intervention); (2) outer setting (ie, the economic, political, and social context within which an organization exists); (3) inner setting (ie, the organizational setting); (4) characteristics of individuals involved in implementation; and (5) the implementation process) (Table 2 and Table 3). Stakeholders included 7 parents of youth ages 12 to 24 years, 7 physicians, 7 nurses and nurse practitioners, 7 leaders of pediatric primary care practices, 6 leaders of behavioral health, 7 leaders of quality improvement, 6 system leaders, 4 third-party payers, and 7 members of national credentialing bodies. Among the stakeholder groups, 2 leaders of pediatric primary care practices, 1 physician, and 4 parents owned a firearm; we do not have firearm ownership information from the other 6 stakeholder groups. Of the interviewers, 5 were female, 3 had PhDs, and 3 had advanced graduate training (ie, masters completed or in progress).

**Table 2.** Participant Demographic Characteristics

Variable	No. (%)	
	Total Participants (N = 58)	Parent Participants (n = 7)
Sex		
Male	27 (47)	3 (43)
Female	31 (53)	4 (57)
Ethnicity		
Hispanic and/or Latino	5 (9)	0
Non-Hispanic and/or non-Latino	37 (64)	7 (100)
Prefer not to disclose	1 (2)	0
Missing	15 (26)	0
Race^a^		
American Indian or Alaska Native	1 (2)	0
Asian	4 (7)	0
Black or African American	3 (5)	2 (29)
Native Hawaiian or Pacific Islander	0	0
White	33 (57)	4 (57)
Multiple races	1 (2)	1 (14)
Other	1 (2)	0
Prefer not to disclose	2 (3)	0
Missing	15 (26)	0

^a^ Because participants could select multiple responses, percentages may not sum to 100.

**Table 3.** Qualitative Interview Themes and Quotes

Theme	Example Quotes
Outer setting (patient characteristics; prevalence of firearms; firearm culture)	Behavioral health leader 1:“So we’re talking about coming into a culture trying to do a very reasonable urban intervention on a mostly rural population that is politically very, very, very charged around gun rights.”
	Leader of primary care practice 1: “Some [clinicians] might be concerned that some of their parents would take it…take it personally or take it badly.”
	Physician 1: “Most of my patients don’t seem threatened because I don’t approach it [discussions about firearm ownership] in a very threatening manner.”
Inner setting (health system involvement; leadership support; practice variability; clinician turnover)	Leader of primary care practice 2: “I think the [health system] is really good about standardizing things, and rolling it out…But at that top level, if that level is not sold on it, then nothing will happen.”
	Physician 2: “So in terms of how things operate we usually have to get it [a new intervention] approved from the system and once it’s approved from the system, it gets rolled out to the regional person in charge and they educate the smaller group.”
Characteristics of the individuals (confidence/self-efficacy; training and education needs; clinician comfort and approach; roles and responsibilities)	Leader of primary care practice 3: “I don’t see any resistance [to implementing firearm safety check] from any of my clinicians.”
	Physician 3: “I would say most physicians are comfortable with asking about firearms.”
	Physician 4: “I don’t think our staff knows much about gun safety.”
	Parent 1: “I think the doctor would need to have some background as to why people need or want guns and not be judgmental.”
Intervention characteristics (acceptability; feasibility; importance of brevity; training and education needs; financing, storing, and distributing locks; liability concerns)	Leader of primary care practice 4: “It has to be something very concise, very to-the-point that does take, you know, ideally no more than a minute, so we can implement it. I think that’s the way a good intervention is set up. So you screen to see if it’s pertinent and then, if it is, then you counsel them on it, and then if…you know after you’ve done the counseling, to actually give them something that’s useful to help drive that home of a gun lock is, I mean, the way it should be. So I wouldn’t make any changes.”
	Leader of primary care practice 3: “I think that gun locks are probably the best option, but I’m not sure how many people would be willing to actually go for it.”
Barriers (time; clinician resistance to change; cost; storage constrains; firearm culture; liability concerns; clinician turnover; clinician comfort and self-efficacy)	Nurse 1: “Time is the only barrier that I can think of that we run into when we add kind of new things into the clinic work flow.”
	Leader of primary care practice 4: “I don’t know what they [firearm locks] cost and I don’t think that that would necessarily be something that we would be able to invest in.”
	Leader of primary care practice 1: “Some might be concerned that some of their parents would take it…take it personally or take it badly.”
	Physician 5: “I wonder if somebody would say, ‘Wait a second. Now we give out gun locks? Is there any liability associated with that if somebody doesn’t use them?’”
Facilitators (clinician and parent openness; intervention setting fit; existing infrastructure; consistent with priorities; would be supported by key opinion leaders)	Nurse 2: “I definitely think it’s appropriate for the primary care providers and any primary care—the physicians and anyone working from the team in the primary care clinic—to be asking about weapons in the home.”
	System leader 1: “I think that certainly primary care plays a central role in suicide prevention.”
	Parent 2: “I just feel as long as they’re [physicians] educated on gun laws and prevention, I would be okay with it.”
Implementation strategies (integrate with electronic health record; administer surveys; provide written materials; screen and distribute locks out of examination room; implement as universal intervention during well visits and bundle with other safety screening; pilot before deployment; training and education; marketing; use a policy mandate; creative financing)	Leader of primary care practice 2: “If you really want things done, you put it there [electronic health record] and then it’s easy to track whether or not they did it.”
	Leader of primary care practice 1: “Have written materials that they can hand out…I think it would be helpful to have some scenarios where we try to anticipate what people’s responses might be.”
	Leader of primary care practice 3: “We might start by seeing if we can just implement adding that question [screening for firearms in the home] to our basic questions that we ask when the kid comes in for a well check.”
	Physician 4: “We’d put a policy around it so that everybody’s aware.”

### The CFIR Informed Themes

#### Outer Setting

Participants noted that most patients and families would likely be receptive to Firearm Safety Check. Patients served included individuals in the inner city who may have firearms for protection in areas with high gun violence, and suburban and rural populations who may own firearms for hunting or recreational purposes. There was a perception that some, especially those in inner cities, may be unable to afford locks and thus more receptive to the Firearm Safety Check intervention than others. In general, physicians practicing in the inner city and suburbs thought the intervention would be perceived as more acceptable by patients than those in rural areas. However, it was also noted that those who keep firearms for protection might be less likely to use locks in order to keep firearms accessible. Overall, firearm ownership was described as prevalent in the communities served by both health systems. Many respondents noted that Firearm Safety Check would be consistent with recommendations made by relevant organizations, and representatives from national organizations and credentialing bodies expressed a willingness to support efforts integrating the intervention into routine practice.

#### Firearm Culture

Many participants reported that US firearm culture would be important in the implementation of Firearm Safety Check*.* Physicians and leaders noted that some patients and families may feel firearm ownership screening infringes on their Second Amendment rights. These concerns were especially prevalent among participants from the Southern site. Clinicians also raised concerns that parents may not honestly disclose firearm ownership owing to either privacy concerns or illegal ownership of the firearm. Clinicians reported largely knowing that they had the legal right to ask about firearm ownership and to discuss safe storage with patients and families.^15^ Finally, it was also reported that recent high-profile gun-related incidents, such as mass shootings, have increased awareness, making these conversations easier for clinicians to initiate.

#### Inner Setting

Participants noted that new programs should be implemented systemwide with approval and support of health system leadership. In some instances, leaders of practices felt they could implement Firearm Safety Check on their own; however, if the implementation was system led, it would be easier to obtain funding (eg, to pay for firearm locks) or support integration into the electronic health record (EHR). Participants noted that in large health systems it could be difficult for clinicians on the ground to navigate new intervention approval owing to the bureaucracy involved. Interventions consistent with key system priorities, such as patient safety, may be more likely to obtain system leadership support. Typically, it was described that new programs are usually initiated by leadership and then disseminated through email or during practicewide clinician meetings. It was noted that once implementation moves from the system to the practices, there likely will be between-clinic variability in implementation fidelity or idiosyncrasies to be worked through across sites (eg, residency clinics have unique considerations). Finally, participants noted that clinician turnover occurs with frequency and must be incorporated into implementation planning.

### Characteristics of the Individuals

Clinicians varied in their confidence to implement Firearm Safety Check*.* Some clinicians stated they lacked expertise around firearm locks and wanted additional training. While many clinicians said that it was important and within their scope of practice to facilitate conversations about firearms, some reported feeling uncomfortable with this. When parents were asked about characteristics they would like to see in implementers, most stated they wanted the clinician to take a nonjudgmental stance.

With regard to implementers, there were some nuances described by type of clinic or clinician. It was generally noted that medical assistants or nursing staff would likely screen patients for firearm ownership and distribute locks, while physicians would offer counseling during wellness visits. Practice or health system leaders were reported to likely be involved in decisions to implement the intervention. Other pertinent individuals were those associated with the EHR development and modification and reporting (eg, to develop tools such as Smart Sets).

### Intervention Characteristics

Overall, participants reported that they found Firearm Safety Check highly acceptable. When asked what the intervention should be called, most respondents reported they liked the proposed name of Firearm Safety Check or proposed something similar (eg, Firearm Safety). Participants were also asked separately about each intervention component.

#### Screening

Most individuals, including clinicians and parents, reported screening about firearm ownership was acceptable and would be feasible to implement, particularly if brief, implemented during wellness visits, and bundled with other screening questions (eg, bike helmet usage). Clinicians requested brief training around best screening practices prior to implementation. Suggestions for streamlining screening included administering questions by paper and pencil or on waiting room tablets, or by sending them to families in advance through secure EHR-supported mechanisms. The importance of embedding screening into the EHR was frequently noted.

#### Counseling

Clinicians suggested that counseling around safe firearm storage must be brief, which could be supported by providing resources (eg, scripts). Most participants agreed that if the counseling lasted 1 minute or less it would be feasible.

#### Firearm Locks

Of the intervention components, firearm locks were described as the most difficult to implement owing to concerns about financing, storage, and distribution. No clinicians reported having experience distributing firearm locks to patients. A number of clinicians noted a particular concern about the expectation of teaching patients and families how to use firearm locks properly, possibly owing to liability concerns. Another concern was that people who do not already store their firearms securely will not use the free locks (ie, they perceived individuals would already be storing their firearms securely if they were interested in doing so). Some participants reported that they would prefer clinicians refer them to places to get free locks in the community vs distributing them in the office (eg, by partnering with police). Most parents reported that they would be receptive to receiving locks in pediatric primary care, with the preference of being asked how many locks they would like vs if they would like one, and being asked face to face by their child’s primary care physician and not another staff member.

### Additional Themes That Emerged

#### Barriers

Time was the most frequently noted barrier to implementation. It was reported that clinicians already have too much to do, so adding any new intervention would be challenging. Time to get a new intervention approved by leadership was also cited as a barrier. Additionally, it was noted that some clinicians would be resistant to changing their behavior.

#### Facilitators

A number of facilitators were identified. Clinicians expressed that they would be open to engaging in the Firearm Safety Check, as it fit well within the scope of pediatric primary care and current system priorities. Existing infrastructure that could support launching Firearm Safety Check included established EHR systems and standardized training procedures and policies. The health systems were already doing some mental health and suicide screening, so Firearm Safety Check was seen as complementary. Parents reported being open to having conversations about firearm storage despite acknowledging that their child’s clinician had not previously discussed it.

#### Post Hoc Analysis

Overall, themes did not differ by respondent type when comparing responses. Firearm owner responses were largely consistent with nonfirearm owners, and drew on actual experience with firearms. Firearm owners tended to reinforce the idea that people who own firearms will already be storing them safely. Parent responses were consistent with nonparents. A minor difference was that parents were more likely to voice concerns about financial burdens of firearm locks, reinforcing the importance of giving out actual safe storage devices.

### Implementation Strategies

Participants provided suggestions for implementing Firearm Safety Check in pediatric primary care. The importance of integrating the intervention into the EHR was frequently noted. This was recommended both for ease (eg, triggering reminders about screening) and for ongoing monitoring. Another suggested strategy for monitoring use and satisfaction was to administer clinician and patient surveys. Hard-copy materials, such as informational pamphlets in English and Spanish, were suggested. To lower clinician burden, it was suggested screening could be conducted outside of the examination room (eg, in the waiting room) prior to the visit and that locks could be distributed afterward (eg, by front desk staff). Most respondents suggested implementing Firearm Safety Check as a universal intervention during wellness visits, bundled with other safety questions. Piloting the intervention before wide-scale deployment was also suggested.

Staff education and training on how to deliver the intervention was frequently suggested, noting this could likely be conducted within the existing practice structure (eg, during monthly clinician meetings). Marketing the intervention to clinicians and patients was also suggested, as was the need for clinicians to communicate to families the universal nature of the intervention to avoid the perception of stigmatizing youths by singling them out as at risk.

Additional implementation strategies identified included a policy mandate and exploring creative financing. These included partnering with local police, gun shops, and/or firearm safety programs, applying for grants, working with private payers and insurers to cover the cost of locks, and private donations.

## Discussion

A primary objective of this project was to understand important factors in implementing an evidence-based practice for firearm safety promotion in pediatric primary care as a universal suicide prevention strategy. We solicited perspectives from stakeholders who would be affected by implementation within 2 large health systems.^21^ The results provided valuable insights toward the design of implementation strategies in future efforts to implement firearm safety promotion aligning with recommendations around implementation strategy design with an eye toward targets and mechanisms.^16^

Broadly, health system stakeholders indicated that firearm safety promotion is a priority. This suggests a shift in the way that clinicians think about their role in firearm safety and recognition that asking about firearm ownership and counseling patients about firearm safety are within scope of practice.^22,23^ Participants across stakeholder groups noted the need for a national conversation about firearm safety with all sides represented. One potential way to ensure that firearm safety interventions respect needs across a variety of stakeholders is to take a community-partnered approach. For example, an ongoing study by Barber and colleagues^24^ exemplifies this approach in that gun shop owners and firearm instructors are partnering with researchers with a shared agenda of firearm safety. This innovative work provides a promising model.

Important to this line of inquiry was obtaining a better understanding of the intervention’s acceptability to stakeholders, both firearm owning and not. Generally, firearm-owning and non–firearm-owning stakeholders were consistent with one another in their perceptions of the intervention’s feasibility and acceptability. The stakeholders interviewed were largely supportive of Firearm Safety Check, although the distribution of firearm locks proved to be the most complicated to implement and where additional adaptations may be needed. For example, firearm cabinets may be more acceptable than cable locks.^25^ This underscores the importance of asking stakeholders about individual intervention components vs entire intervention packages.^26^ Fortunately, an established literature exists to guide rigorous intervention adaptations.^27^ We did not query around beliefs of intervention effectiveness; this is a topic worthy of future research.

This work also highlights the importance of considering how best to support clinicians and improve their self-efficacy. One-time trainings are insufficient and ongoing support is important.^28,29^ For example, ongoing consultation or leveraging existing mental health screening or suicide prevention approaches may prove useful. The implementation strategies identified here provide promising approaches to supporting implementation of Firearm Safety Check. In particular, stakeholders noted that it will be important to thoughtfully leverage existing infrastructures, such as EHRs, and to offer brief counseling.^30^ Models of practice that integrate behavioral health and primary care may support the implementation of Firearm Safety Check in concert with other suicide prevention techniques (ie, screening for depression and suicidal ideation) without adding undue burden to clinicians.^31^

### Limitations

While this study is strengthened by its inclusion of diverse stakeholders across multiple systems and a theory-informed interview approach, several limitations should be noted. First, participants reported how they believed the intervention would or could be implemented. They did not necessarily have familiarity with implementing Firearm Safety Check, although many had experience asking parents about firearm ownership and counseling them around safe storage. Second, we did not systematically collect information from stakeholders on whether they represented suburban, urban, or rural perspectives, although both health systems spanned these settings. Third, we did not focus our sampling on physicians and leaders of practices who reported not having used any components of the intervention. Fourth, the study’s subsample of firearm owners was small. Additionally, we did not include stakeholders such as firearm instructors or gun shop owners.

## Conclusions

To our knowledge, this is the first study to report preimplementation contextual inquiry for a universal suicide prevention approach in pediatric primary care. This comprehensive project is innovative in its participatory approach and inclusion of multiple stakeholder perspectives prior to implementation. It is our hope that our findings will aid in developing multilevel implementation strategies to change clinician, organization, and system behaviors around firearm safety promotion in pediatric primary care.
